# Optimisation of the *Schizosaccharomyces pombe urg1* Expression System

**DOI:** 10.1371/journal.pone.0083800

**Published:** 2013-12-20

**Authors:** Adam T. Watson, Yasukazu Daigaku, Saed Mohebi, Thomas J. Etheridge, Charly Chahwan, Johanne M. Murray, Antony M. Carr

**Affiliations:** Genome Damage and Stability Centre, School of Life Sciences, University of Sussex, Brighton, East Sussex, United Kingdom; Universita' di Milano, Italy

## Abstract

The ability to study protein function *in vivo* often relies on systems that regulate the presence and absence of the protein of interest. Two limitations for previously described transcriptional control systems that are used to regulate protein expression in fission yeast are: the time taken for inducing conditions to initiate transcription and the ability to achieve very low basal transcription in the “OFF-state”. In previous work, we described a Cre recombination-mediated system that allows the rapid and efficient regulation of any gene of interest by the *urg1* promoter, which has a dynamic range of approximately 75-fold and which is induced within 30-60 minutes of uracil addition. In this report we describe easy-to-use and versatile modules that can be exploited to significantly tune down P*_urg1_* “OFF-levels” while maintaining an equivalent dynamic range. We also provide plasmids and tools for combining P*_urg1_* transcriptional control with the auxin degron tag to help maintain a null-like phenotype. We demonstrate the utility of this system by improved regulation of HO-dependent site-specific DSB formation, by the regulation Rtf1-dependent replication fork arrest and by controlling Rhp18^Rad18^-dependent post replication repair.

## Introduction

The study of protein function *in vivo* is greatly aided by systems that deplete the protein of interest. Whether or not depletion of a protein is biologically significant (causes a phenotype) will depend on the amount of protein required for its function. The amount of cellular protein is the result of multiple levels of regulation, including transcription rate, mRNA stability, translational efficiency and protein turnover. The study of gene function may require control over one or more of these processes.

In the fission yeast *Schizosaccharomyces pombe* transcription rate has traditionally been controlled using modified constitutive or inducible promoters of varying strength. The promoter of the alcohol dehydrogenase (*adh1^+^*) gene, it’s weak derivative *adh1-*15 and it’s much weaker derivative *adh1-*81, are typical examples of a widely used constitutive promoter [1], [2], [3]. The most widely used inducible promoter used in *S. pombe* is derived from the *nmt1^+^* (no message in thiamine) gene [4]. The *nmt1* promoter has the added advantage of intermediate promoter strengths that are achieved through mutation of the TATA box, generating intermediate (*nmt41*) and low (*nmt81*) strength versions [5].

**Figure 1:**
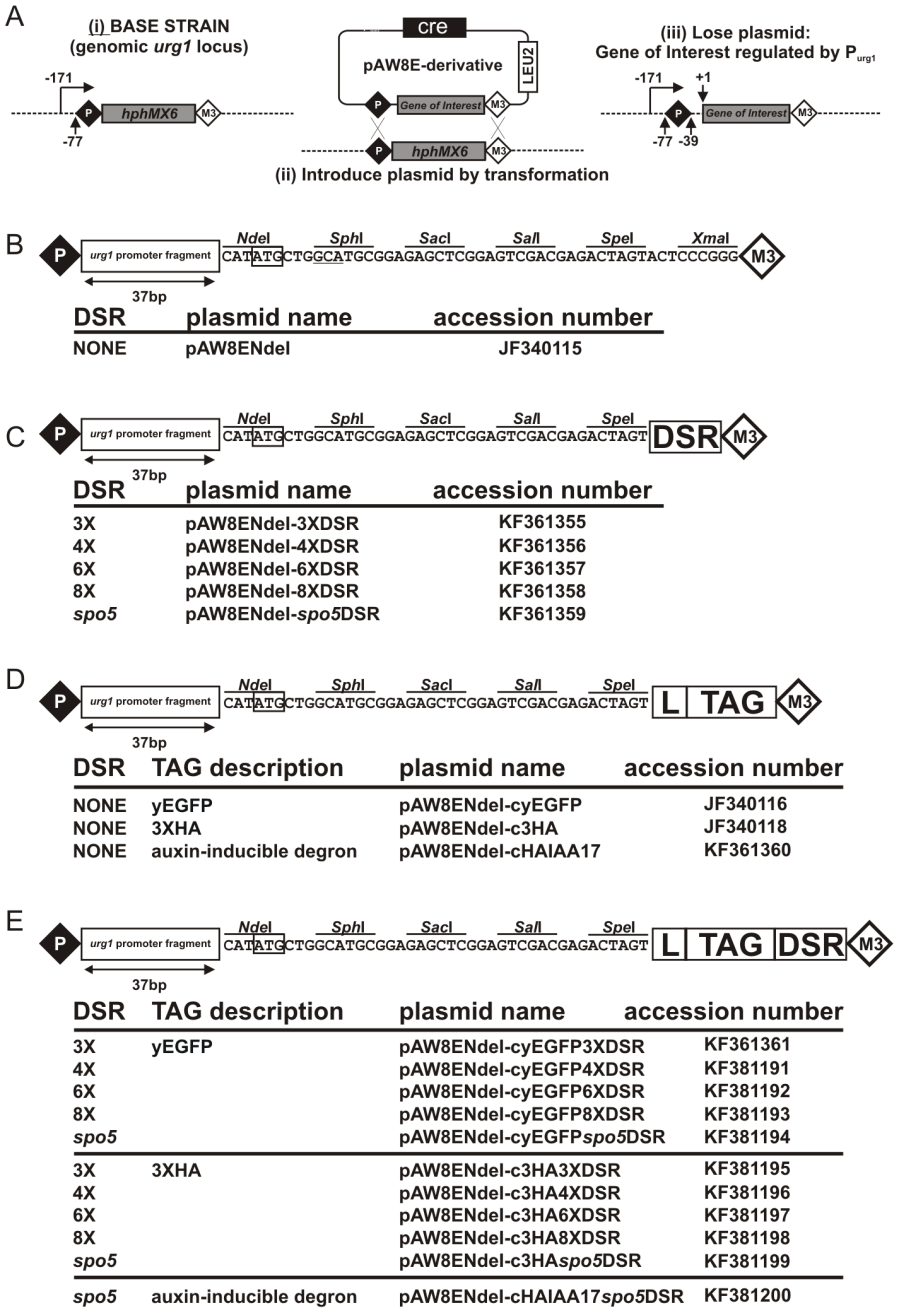
Principals of RCME and plasmids created. (**A**). Schematic showing the process of RCME (Watson 2008): (i) starting with a base strain in which the *urg1* ORF is replaced by an antibiotic marker (each of *hphMX6*, *natMX6* and *kanMX6* are available) that is flanked by (incompatible) loxP and loxM3 sites, a plasmid (ii) is introduced. This plasmid contains the cloned gene of interest and any tagging sequences positioned between loxP and loxM3 sites. It also expresses Cre recombinase. Site-directed recombination next exchanges the sequences between the plasmid and the chromosome (iii). Successful exchange can easily be identified by loss of the antibiotic marker, typically seen in greater than 50% of cells. Plasmid loss in these colonies is then confirmed by replica plating to verify colonies are leu^−^. In our experience, all of these are successful integrants. (**B**). Plasmid for expression of untagged sequences (NO DSR) as previously published [7]. Shown is a schematic of the sequence between loxP (P) and loxM3 (M3) for pAW8E*Nde*I. A start codon is formed from an *Nde*I site. (**C**) Equivalent schematic of pAW8E*Nde*I containing various DSR sequences. (**D**) Schematic of plasmid used to express proteins with either a yEGFP tag, a 3xHA tag or an HA combined with an IAA17 degron tag (HAIAA17) (all with NO DSR). L  =  poly-tyrosine–glycine–serine (TGS) linker: TAG  =  yEGFP, 3xHA or HAIAA17 protein tag. (**E**) Equivalent schematic of pAW8E*Nde*I C-terminal tagging plasmids that also contain various DSR sequences. HA  =  human influenza hemagglutinin protein tag, yEGFP  =  yeast codon optimised green fluorescent protein, HAIAA17  =  Degron from Arabidopsis thaliana transcription repressor.

While these *nmt*-derived promoters offer a choice of transcription levels, they all take 12-16 hours to show induction and 15–21 hours to reach maximum induction levels once thiamine is removed. This is a significant disadvantage considering that the fission yeast cell cycle is completed within 2–3 hours. More recently, Watt et al, (2008) characterised the promoter of the *urg1^+^* gene, where *urg1* transcript levels peak 30 minutes after the addition of uracil [6]. However, attempts to reproduce this ectopically resulted in a significant increase in “OFF-state” transcription, severely limiting the dynamic range and thus its utility. We recently demonstrated that induction kinetics driven by the *urg1* promoter (P*_urg1_*), and the dynamic range, are maintained when ectopic open reading frames (ORFs) replace the native *urg1* ORF [7]. To facilitate rapid and simple manipulation of *urg1* locus, a Cre recombinase and lox recombination site-based Recombination-Mediated Cassette Exchange (RMCE) system was developed [7]. This facilitates rapid and efficient exchange of sequences to place any chosen ORF under control of the endogenous P*_urg1_* (for schematic, see Figure 1A).

While it is now possible to regulate transcription of any gene at the *urg1* locus in response to uracil addition, several disadvantages remain: first, while the dynamic range of ∼75 fold is good, the basal level of proteins regulated by P*_urg1_* remain significant. Thus, the minimal repressed (“OFF”) level of protein is often too high to visualise a phenotype equivalent to a null mutation. Second, the induced (“ON”) level is correspondingly also high, which may cause problems when studying proteins who’s endogenous levels are comparatively low. Third, while P*_urg1_* transcription resets to basal levels within 30–60 minutes of uracil removal, the protein being studied will decay with kinetics that are determined by the stability of the protein produced. We thus sought mechanisms to further regulate P*_urg1_*-dependent transcript levels and to facilitate removal of the induced protein following transcription shut-off.

In *S. pombe*, a mechanism for the selective removal of meiosis-specific mRNAs in mitotic cells has been characterised. The removal involves the YTH-domain containing protein Mmi1 [8] which binds meiosis-specific mRNAs containing Determinant of Selective Removal (DSR) sequences, usually located at the 3’ end of the transcript. Mmi1 greatly increases transcript turnover by directing DSR-containing transcripts to nuclear exosomes for degradation [8], [9]. By deletion analysis, the DSR elements of *ssm4*, *rec8* and *spo5* mRNAs were identified [8]. Recent studies have recognised a hexanucleotide motif U(U/C)AAAC that is highly enriched in the DSR elements and have shown that tandem repeats of this motif can function as an artificial DSR in heterologous gene systems [10], [11].

Recent studies in *S. pombe* indicate that ∼1 mRNA copy per cell defines a functional norm for productive gene expression [12]. Since aggregate levels of significantly less than 1 transcript per cell will provide a distribution of 0 or 1 transcripts in most of the cells in the population, this may help explain why it is difficult to obtain "null" phenotypes using transcriptional control alone: individual cells in the population will be producing significant quantities of protein, even when the aggregate transcript level appears very low. Furthermore, even if a transcript is “shut-off” completely, the intrinsic stability of the protein expressed will determine how quickly a null phenotype will be established when “shut-off” experiments are performed.

Protein levels can be manipulated independently of transcription using various protein degradation systems. These generally involve the fusion of a domain (known as a ‘degron’) to the target protein to induce degradation. The auxin-inducible degron (AID) system [13] was recently adapted for use in fission yeast [1]. In plants, the hormone auxin bind to the transport inhibitor response 1 (TIR1) F-box protein and promotes binding of the E3 ubiquitin ligase SCF^TIR1^, an SCF (Skp1, Cullin and F-box) ubiquitin ligase complex, to auxin or IAA (Aux/IAA) transcription repressors [14]. The Aux/IAA proteins are subsequently poly-ubiquitinated by SCF^TIR1^ and degraded by the proteasome. All eukaryotes have multiple subtypes of the SCF ubiquitin ligases, but orthologs of TIR1 and Aux/IAAs are only found in plants. The degradation system described by Nishimura et al. (2009) for budding yeast uses the IAA17 degron from Arabidopsis thaliana. When fused to a protein target, this degron sequence promotes proteasome and ubiquitin-dependent degradation in an auxin-dependent manner if a functional TIR1 F-box protein is also concomitantly expressed. Rapid depletion of a target protein within 30 mins in the presence of auxin was observed, allowing the generation of conditional mutants [13]. The AID system has also been adapted for *S. pombe*, but appears to be somewhat less efficient. However, auxin-dependent conditional mutant phenotypes were obtained for several proteins when the corresponding protein was tagged with IAA17, a TIR1-Skp1 fusion protein was expressed and the system was combined with transcription repression [1].

Here we describe for *S. pombe* a P*_urg1_*-based, uracil regulatable protein expression system that exploits control over the combination of transcription rate, mRNA stability and protein-depletion to tightly control target protein expression levels in *S. pombe*. The rapid induction of the *urg1* ^+^ promoter controls transcription rate, DSR sequences regulate transcript levels via constitutive mRNA degradation and the auxin-inducible protein degradation system controls protein turnover. To facilitate a choice of “ON” and “OFF” levels we have constructed a range of plasmid vectors that allow researchers to use RMCE to rapidly and efficiently insert their gene of interest at the *urg1* locus in the context of the desired DSR and degron sequences (Figure 1). The plasmids contain a variety of DSR constructs to determine different levels of transcript stability and further allow the cloned ORF to be untagged or tagged with yeast codon-optimised green fluorescent protein yEGFP, the hemagglutinin epitope tag HA as well as the auxin-inducible IAA17 degron.

## Results

### The Mmi1/DSR mRNA degradation system reduces protein levels expressed from the *urg1* locus

An advantage of the *S. pombe nmt1* (*no message in thiamine*) inducible promoter system is the ability to attenuate promoter activity levels through progressive deletion of the TATAA-box sequence [5]. However, deletion of a potential *urg1* promoter TATAA box sequence identified by 5’ RACE [7] did not significantly reduce promoter activity (data not shown). We therefore decided to exploit the recently characterised mechanism that selectively removes meiosis-specific mRNAs from mitotic cells in *S. pombe*. The mechanism involves Mmi1 binding to a target region – the DSR (Determinant of Selective Removal) and guiding the mRNA for degradation via the nuclear exosomes [8], [9]. The hexanucleotide motif U(U/C)AAAC is highly enriched in the DSR and tandem repeats of this motif function as an artificial DSR in heterologous gene systems [10], [11].

**Figure 2:**
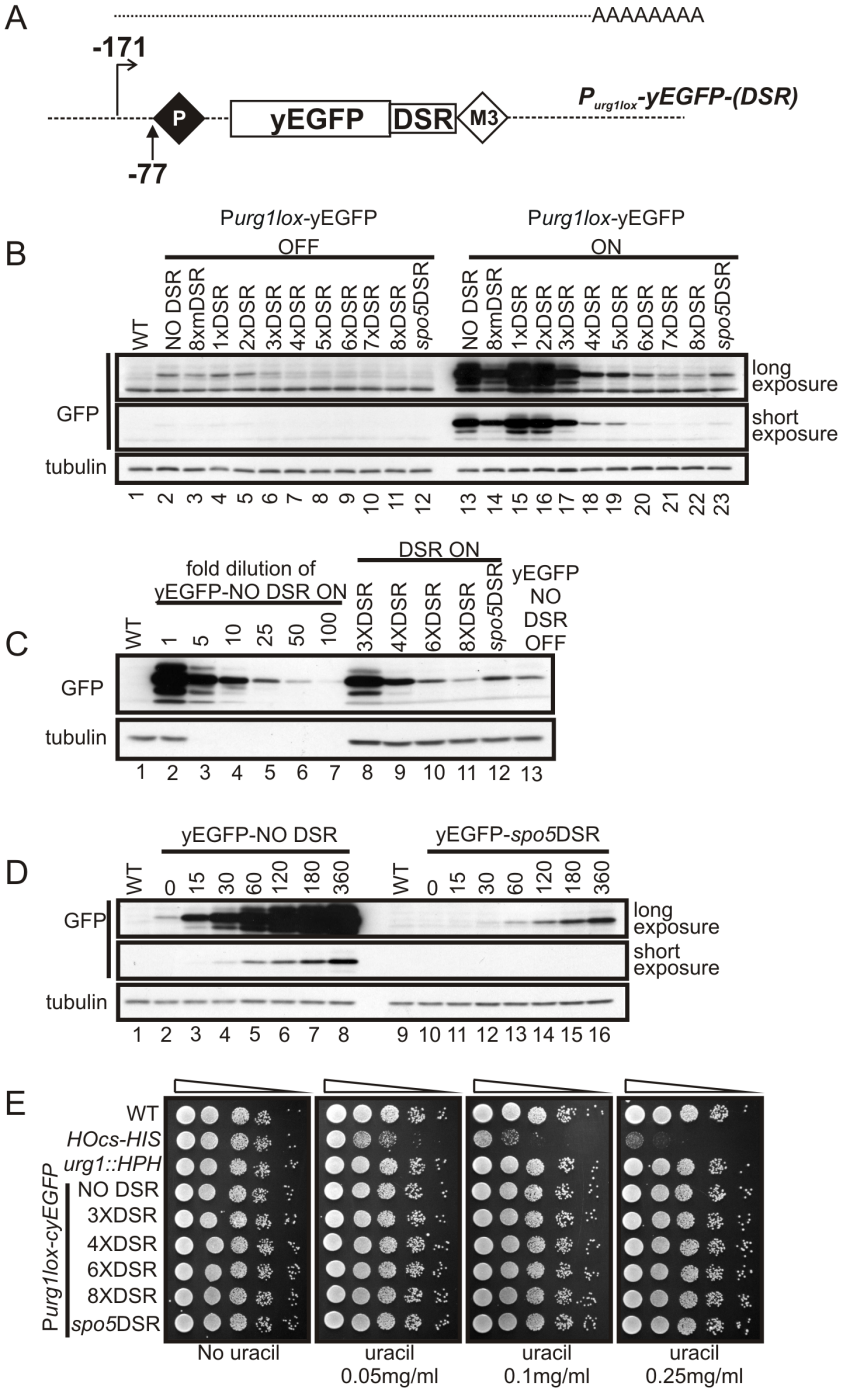
Determinant of Selective Removal (DSR) sequences can reduce protein levels expressed from P*_urg1lox_*. (**A**). Schematic of the P*_urg1lox_* locus following RCME to introduce yEGFP under the control of the *urg1* promoter. The resulting transcript encodes the ORF followed immediately after the stop codon by one of a variety of DSR sequences. These act to target the transcript to the nuclear exosome. (**B**). The strains created with the yEGFP ORF with and without DSR sequences present in the 3’ UTR: AW640 (NO DSR), AW702 (8XmDSR), AW726 (1XDSR), AW728 (2XDSR), AW730 (3XDSR), AW732 (4XDSR), AW694 (5XDSR), AW696 (6XDSR), AW698 (7XDSR), AW700 (8XDSR) and AW638 (*spo5*DSR) (see also Table 1). The strains were cultured in EMM+L to ∼5×10^6^ cells at 30°C (uracil absent - P*_urg1lox_* OFF). Uracil was added at 0.25 mg/ml and cells grown for 120 minutes (P*_urag1lox_* ON). Total protein extracts from un-induced P*_urg1lox_* OFF cells (lanes 2–12) and induced P*_urg1lox_* ON cells (lanes 13–23) were separated by SDS PAGE prior to Western blotting using anti-GFP to detect yEGFP (upper panels) and anti-tubulin for a loading control (lower panel). Lane 1  =  WT control *urg1* ^+^ strain (AW501). (**C**) Comparison of the induced P*_urg1lox_*-yEGFP levels in B. Induced cell total protein extracts were used to estimate the fold-decrease in protein levels after induction. The NO DSR sample (lane 13 panel B) was serially diluted using SDS sample buffer and analysed (lanes 2 to 7) alongside undiluted induced 3XDSR (AW730 - lane 8), 4XDSR (AW732 - lane 9), 6XDSR (AW696 - lane 10), 8XDSR (AW700 - lane 11) and *spo5*DSR (AW638 - lane 12) samples. Lane 13  =  undiluted un-induced NO DSR sample (the same as lane 2 panel B). (**D**) The kinetics of yEGFP accumulation is unaffected when using a DSR element. Time-course showing yEGFP protein levels in NO DSR (AW640 - lanes 2 to 8) and *spo5*DSR (AW638 - lanes 10 to 16) cells after addition of uracil at 0.25 mg/ml to induce P*_urg1lox_*. Analysis of yEGFP levels by western blot as described in B. Samples taken at time-points shown (mins). Lanes 1 and 9  =  control *urg1* ^+^ strain (AW501). (**E**) Over-expression of mRNAs containing DSR sequences does not affect cells growth/viability. Strains shown were serially diluted 10-fold in water and spotted on EMM+L plates supplemented with uracil at concentration shown. Pictures were taken after 3 days at 30°C.

We modified our published *urg1* promoter system [7] to contain either the 157bp DSR element derived from the *S. pombe spo5* gene or various numbers of repeats of the DSR core motif: TTAAAC. To achieve this, we modified the Cre-expression plasmid pAW8E*Nde*I [7] by inserting either the 157bp *spo5*DSR element or between 1 and 8 copies (referred to as 1XDSR through 8XDSR) of the DSR core motif adjacent to the pAW8E*Nde*I multiple cloning site such that, in the corresponding mRNA, it will be 3’ of the ORF. To maintain identical motif spacing to that previously characterised [10], the repeat motifs were separated by six base pairs copied randomly from bacteriophage lambda DNA. We next introduced the open reading frame (ORF) of the yeast codon-optimised green fluorescent protein (yEGFP) between the MCS and the DSR sequences. When integrated at the *urg1* locus, these constructs will express GFP as the ATG initiation codon contained within the *Nde*I restriction enzyme site (CATATG), present in pAW8E*Nde*I MCS, is in-frame with the yEGFP ORF. These pAW8E*NdeI*-cyEGFP-DSR plasmids were then used to create, via Cre-mediated RMCE, a series of yeast strains where expression of yEGFP was controlled at the *urg1* locus by the P*_urg1lox_* (we designate the modified *urg1* promoter, which remains at the *urg1* locus, but contains a loxP recombination site at –37, P*_urg1lox_* to distinguish it from the endogenous promoter). The transcripts resulting from the P*_urg1lox_* in these cells contain, in their 3’ untranslated regions, various forms of the DSR (Figure 2A). A control strain containing no DSR sequences (NO DSR) and another carrying 8 copies of the mutated core motif GTAAAC (8XmDSR) were also constructed. This mutated core motif has been shown to largely ablate DSR activity [10].

To examine how efficiently the DSR/Mmi1 RNA degradation pathway reduced yEGFP protein levels, the amount of yEGFP in extracts prepared from log-phase cells in the absence (P*_urg1lox_* OFF) or presence (P*_urg1lox_* ON) of uracil were analysed by immunoblotting (Figure 2B). The *P_urg1lox_-yEGFP*-*NO DSR* cells accumulated high levels of yEGFP protein 120mins after induction (lane 13) but a clear signal was also observable in un-induced *P_urg1lox_-yEGFP*-*NO DSR* cells when compared to control AW501 (*urg1^+^*) cells (lanes 2 and 1 respectively). This demonstrates the rapid induction of the *urg1* system and also the “leakiness” of the repressed *urg1* promoter. One and two copies of the DSR core motif (TTAAAC) show no significant reduction of yEGFP protein levels either in un-induced (lanes 4 and 5) or induced cells (lanes 15 and 16). However, increasing the number of core motifs to three (*P_urg1lox_-yEGFP*-*3XDSR* cells) resulted in an observable reduction in protein levels for both un-induced (lane 6) and induced (lane 17) situations. Further reductions in yEGFP expression levels were observed with increasing number of DSR repeats (lanes 18 to 22) indicating increased RNA processing.

*P_urg1lox_-yEGFP*-*8mXDSR* cells, our negative control which harbours eight copies of the mutated (GTAAAC) motif, accumulated significant levels of yEGFP protein (lanes 3 and 14) although there was a modest decrease when compared to the NO DSR control. This implies the mutated motif retains some function. In our positive control cells, *P_urg1lox_-yEGFP*-*spo5DSR*, the amount of yEGFP was significantly reduced following induction by uracil addition. In the un-induced cell samples the detection limit of our western blot analysis was insufficient to show yEGFP levels for *P_urg1lox_-yEGFP*-*4XDSR* through *P_urg1lox_-yEGFP*-*8XDSR* and *P_urg1lox_-yEGFP*-*spo5DSR* cells (lanes 7 to 12). Thus, for these cells we require a more sensitive method to determine P*_urg1lox_* repressed protein levels (see below). Induced *P_urg1lox_-yEGFP*-*spo5DSR* cells showed similar yEGFP levels to those observed for *P_urg1lox_-yEGFP*-*6XDSR* cells (lanes 20 and 23). The relationship between the number of core TTAAAC repeats and DSR activity was not, however, linear (Figure 2B). This may reflect the mechanism whereby Mmi1 binds the DSR containing transcript.

To estimate the fold decrease in induced protein levels we used the samples from Figure 2B for further western blot analysis. The induced *P_urg1lox_-yEGFP*-*NO DSR* sample was serially diluted in SDS-sample buffer 5-, 10-, 25-, 50- and 100-fold (lanes 2 to 7) and immunoblotted along with undiluted induced *P_urg1lox_-yEGFP*-*3XDSR, -4XDSR, -6XDSR, -8XDSR* and *-spo5DSR* cell samples (lanes 8-12, Figure 2C). Three tandem repeats of the DSR core element reduce levels approximately 5-fold when compared to cells containing no DSR sequences. Cells carrying 4, 6 and 8 core repeat motifs reduce levels further (10-, 25- and 50-fold lower respectively). In *P_urg1lox_-yEGFP*-*spo5DSR* cells, the level is reduced approximately 25-fold compared to the NO DSR control. Importantly, the yEGFP level in NO DSR repressed cells (lane 13) lies between the induced levels seen for cells containing 8 DSR repeats (lane 11) and the *spo5DSR* (lane 12). Thus, by exploiting the constitutive degradation of transcripts through the introduction of different DSR constructs we can choose “ON” (+ uracil) and “OFF” (- uracil) levels of protein across a significantly better dynamic range when compared to the constitutive *P_urg1lox_* RMCE system.

The rapid induction of the *P_urg1lox_* is the major advantage of the expression system [7]. To establish if this rapid induction is maintained when DSR regulatory sequences are used, we performed an induction time-course and determined yEGFP protein levels by western blotting (Figure 2D). While the *spo5DSR* sequences reduced the total level of yEGFP, the kinetics of yEGFP accumulation in *P_urg1lox_-yEGFP*-*NO DSR* and *P_urg1lox_-yEGFP*-*spo5DSR* remained very similar (compare the long exposure for *spo5*DSR with the short exposure for NO DSR). These data demonstrate that DSR/Mmi1 RNA degradation pathway allows only a small percentage of DSR-containing transcripts to be translated prior to removal. Following the addition of uracil and the induction of P*_urg1lox_* transcription rate, the increased DSR-containing mRNA levels will result in higher translation efficiency and the kinetics of induction are maintained.

It has been shown that the disruption of *mmi1* severely impairs cell growth [8] and we were concerned that over-expression of mRNAs containing DSR sequences may affect cell growth/viability by titrating the available Mmi1 activity. We therefore performed a spot-test, where cells containing DSRs were spotted onto media containing uracil to induce P*_urg1lox_* (Figure 2E). For a positive control, the strain AW507 was used [7]. In this strain (described in more detail in the next section) the expression of HO-endonuclease is under the control of the *urg1* promoter (P*_urg1lox_-HO*) and the cells contain the HO cut site (HOcs) within the *S. pombe his3^+^* selectable marker (*HOcs-HIS*). HO-dependent cleavage of *HOcs-HIS* thus prevents cell growth when the media does not contain histidine. As expected, growth in presence of uracil and absence of histidine lead to P*_urg1lox_-HO*, *HOcs-HIS* cell inviability linked to the concentration of uracil in the growth media (Figure 2E). However, all the DSR containing strains tested grew equally as well on plates either with or without uracil.

Taken together these results show core-repeat and the *spo5* DSR elements retain selective removal activity when inserted into the 3' UTR of mRNAs expressed at the *urg1* locus and that the DSR/Mmi1 RNA degradation system successfully reduced yEGFP levels without affecting the speed of induction or cell viability/growth.

### The Mmi1/DSR mRNA degradation system attenuates HO expression levels

Because the detection limit of western blot analysis, we were unable to estimate the decrease in “OFF” levels of protein in the various repressed DSR cells (see Figure 2B). We therefore attempted to demonstrate lower P*_urg1lox_* OFF protein levels using biological assays. We used the previously described *S. pombe* single-strand annealing (SSA) assay [7]. The SSA strain contains the MATα minimal HO recognition sequence (HOcs) in-frame and within the coding sequence of the *S. pombe his3* ^+^ selectable marker. This construct, flanked by two homologous sequences, is integrated into chromosome 1 (*HOcs-HIS*; [7]). Regulation of the expression level of the HO endonuclease using P*_urg1lox_* induces double strand breaks (DSBs) at HOcs. DSB ends then undergo resection that results in single strand DNA (ssDNA) tails. If the chromosome is cut in G1 or both sister chromosomes are cut following replication of the region, homologous recombination (HR) repair is not an option and resection continues until both regions of homology become single stranded. Once this occurs, the homologous ssDNAs anneal, resulting in the repair of the chromosome at the expense of loss of the intervening sequences. These sequences include the HOcs and the *his3* ^+^ selectable marker. SSA rates can thus be measured by calculating the percentage of histidine auxotrophic cells by plating cells prior to and after induction of P*_urg1lox_*-HO onto histidine containing media and then replica plated onto media lacking histidine. SSA is an efficient repair mechanism, and thus the rate of marker loss reflects the HO expression level.

**Figure 3:**
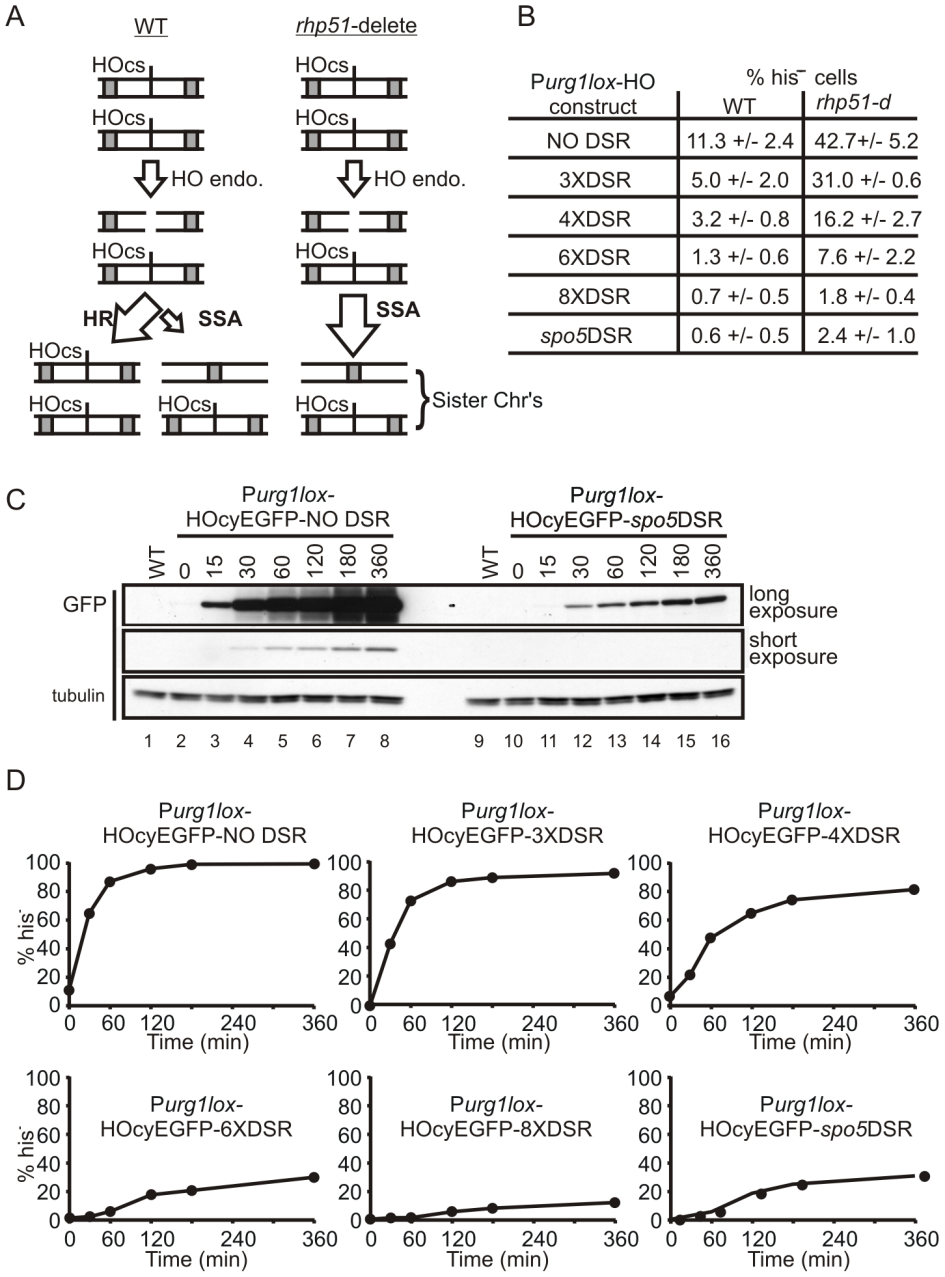
DSR activity reduces both induced and repressed P*_urg1lox_* protein levels. (**A**) Schematic illustration outlining the repair of a single HO-induced DSB in a G2 phase *S. pombe* cell. Repair in a normal (WT) cell can occur by either by homologous recombination (HR) or single strand annealing (SSA) (left panel) whereas repair in an HR deficient *rhp51*-delete cell can only occur via SSA (right panel). Grey box  =  region of homology, HOcs  =  HO endonuclease cut site. (**B**). Steady-state rate of *his3* ^+^ marker loss in WT HR proficient cells compared to HR deficient *rhp51*-delete cells. The HO endonuclease ORF tagged at the C-terminus with yEGFP was inserted by Cre-mediated cassette exchange into the *urg1* locus in WT cells containing the HOcs-HIS construct to create AW741 (NO DSR), AW743 (3XDSR), AW745 (4XDSR), AW747 (6XDSR), AW749 (8XDSR) and AW751 (*spo5*DSR) and in *rhp51*-delete cells to create AW734 (NO DSR), AW816 (3XDSR), AW818 (4XDSR), AW820 (6XDSR), AW822 (8XDSR) and AW739 (*spo5*DSR). Logarithmically growing cells cultured in EMM+L were plated onto EMM+LH plates and grown at 30°C. Colonies were replica plated onto EMM+L plates and the percentage of histidine auxotrophic (his^−^) cells calculated. The assay was repeated at least three times and the average numbers are presented as the mean +/– SD. (**C**) The kinetics of HO-cyEGFP protein accumulation is unaffected by DSR activity. Time-course showing accumulation of HO-cyEGFP protein levels following induction of P*_urg1lox_*. Logarithmically growing AW671 (NO DSR) and AW673 (*spo5*DSR) cells (see Table 1) were induced by the addition of uracil at 0.25mg/ml. HO-cyEGFP protein levels were examined by western blot analysis as described in Figure 2B. Samples taken at time points shown (mins). (**D**) DSR activity slows *his3+* marker loss in the P*_urg1lox_*-HO/HOcs-HIS SSA assay. Strains AW741 (NO DSR), AW743 (3XDSR), AW745 (4XDSR), AW747 (6XDSR), AW749 (8XDSR) and AW751 (*spo5*DSR) were grown in EMM+L to mid-log phase and uracil added at 0.25 mg/ml to induce P*_urg1lox_*. Cells were plated onto EMM+LH plates and grown at 30°C. Colonies were replica plated onto EMM+L plates and the percentage of histidine auxotrophic cells calculated. Samples taken at time-points shown (mins). The assay was repeated twice and numbers shown are the mean. X axis  =  percentage of histidine auxotrophic cells, Y-axis  =  time in minutes.

In *S. pombe*, for >80% of the cell cycle a sister chromatid is present, allowing DSB repair by homologous recombination. When HO is expressed at high levels in *HOcs-HIS* cells, both sister chromatids are likely to be cut in a single cell. However, when HO is expressed at low levels (due to leakiness of the P*_urg1lox_* promoter for example) the DSBs that occur are likely to be formed in only one of the two sister chromatids. This gives the cell the opportunity to use HR to repair the DSB. HR repair from the sister chromosome is silent: it restores both the HOcs site and retains the *his3* ^+^ marker (Figure 3A). When the HR pathway is not available for repair, as in the *rad51*-delete strain, a DSBs on a single sister chromatid can only be repaired using SSA. Following sister segregation at mitosis, one daughter cell will be his^+^ and the other his^−^. Therefore, the rate of marker loss at low levels of HO expression is expected to be higher in *rhp51*-delete cells when compared to WT cells (Figure 3A) and that rate should be a direct reflection of the number of DSBs introduced.

To test the effectiveness of the DSR/Mmi1 pathway, we sub-cloned the HO endonuclease ORF to create Cre-expression plasmids *pAW8ENdeI-HO-yEGFP-NO DSR*, *-3XDSR*, *-4XDSR*, *-6XDSR*, *-8XDSR* and *-spo5DSR*. Using an *urg1* base strain containing the *HOcs-HIS* construct (AW467 – Table 1), the HO-cyEGFP fusion ORF with and without DSR sequences was inserted at the *urg1* locus by cassette exchange. From the resulting strains, the steady-state level of histidine auxotrophic (his^−^) cells in otherwise wild type cells growing logarithmically in the absence of uracil (P*_urg1lox_* OFF) was determined (Figure 3B). For *P_urg1lox_-HO-cyEGFP-NO DSR* cells we observed 11.3 (+/– 2.4)% of cells had lost the *his3* ^+^ marker with all strains carrying DSR elements showing significantly lower levels of marker loss. In cells containing 3 copies of the core DSR motif (*P_urg1lox_-HO-cyEGFP-3XDSR*), the rate was reduced to 5.0 (+/– 2.0)% and increasing number of core motifs furthers decreases the percentage of histidine auxotrophic cells. The *HO-cyEGFP-spo5DSR* cells showed a steady state level of marker loss of 0.6 (+/– 0.5)%, similar to that for *HO-cyEGFP-8XDSR* cells.

**Table 1:** *S. pombe* strains used in this study.

Strains created via Cre-lox recombination mediated cassette exchange during this study		
*urg1* base strain employed	RCME plasmid used	Genotype of strain created
*h-, urg1::RMCE_hphMX6_, leu1-32* (AW459) *(*Watson et al 2011*)*	pAW8E*Nde*I-cyEGFP	*h-, urg1::Purg1lox-cyEGFP, leu1-32 (AW640)*
	pAW8E*Nde*I-cyEGFP-1xDSR	*h-, urg1::Purg1lox-cyEGFP-1xDSR, leu1-32 (AW726)*
	pAW8E*Nde*I-cyEGFP-2xDSR	*h-, urg1::Purg1lox-cyEGFP-2xDSR, leu1-32 (AW728)*
	pAW8E*Nde*I-cyEGFP-3xDSR	*h-, urg1::Purg1lox-cyEGFP-3xDSR, leu1-32 (AW730)*
	pAW8E*Nde*I-cyEGFP-4xDSR	*h-, urg1::Purg1lox-cyEGFP-4xDSR, leu1-32 (AW732)*
	pAW8E*Nde*I-cyEGFP-5xDSR	*h-, urg1::Purg1lox-cyEGFP-5xDSR, leu1-32 (AW694)*
	pAW8E*Nde*I-cyEGFP-6xDSR	*h-, urg1::Purg1lox-cyEGFP-6xDSR, leu1-32 (AW696)*
	pAW8E*Nde*I-cyEGFP-7xDSR	*h-, urg1::Purg1lox-cyEGFP-7xDSR, leu1-32 (AW698)*
	pAW8E*Nde*I-cyEGFP-8xDSR	*h-, urg1::Purg1lox-cyEGFP-8xDSR, leu1-32 (AW700)*
	pAW8E*Nde*I-cyEGFP-8mxDSR	*h-, urg1::Purg1lox-cyEGFP-8mxDSR, leu1-32 (AW702)*
	pAW8E*Nde*I-cyEGFP-*spo5*DSR	*h-, urg1::Purg1lox-yEGFP-spo5DSR, leu1-32 (AW638)*
	pAW8E*Nde*I-HO-cyEGFP	*h-, urg1::Purg1lox-HO-cyEGFP, leu1-32 (AW671)*
	pAW8E*Nde*I-HO-cyEGFP-spo5DSR	*h-, urg1::Purg1lox-HO-cyEGFP-spo5DSR, leu1-32 (AW673)*
*h-, urg1::RMCE_hphMX6_, LEU-HOcs-his3+-λ-EU2, leu1-32, his3-D1 (AW467 Watson et al 2011)*	pAW8E*Nde*I-HO-cyEGFP	*h-, urg1::Purg1lox-HO-cyEGFP, LEU-HOcs-his3+-λ-EU2, leu1-32, his3D1 (AW741)*
	pAW8E*Nde*I-HO-cyEGFP-3XDSR	*h-, urg1::Purg1lox-HO-cyEGFP-3XDSR, LEU-HOcs-his3+-λ-EU2, leu1-32, his3D1 (AW743)*
	pAW8E*Nde*I-HO-cyEGFP-4XDSR	*h-, urg1::Purg1lox-HO-cyEGFP-4XDSR, LEU-HOcs-his3+-λ-EU2, leu1-32, his3D1 (AW745)*
	pAW8E*Nde*I-HO-cyEGFP-6XDSR	*h-, urg1::Purg1lox-HO-cyEGFP-6XDSR, LEU-HOcs-his3+-λ-EU2, leu1-32, his3D1 (AW747)*
	pAW8E*Nde*I-HO-cyEGFP-8XDSR	*h-, urg1::Purg1lox-HO-cyEGFP-8XDSR, LEU-HOcs-his3+-λ-EU2, leu1-32, his3D1 (AW749)*
	pAW8E*Nde*I-HO-cyEGFP-*spo5*DSR	*h-, urg1::Purg1lox-HO-cyEGFP-spo5DSR, LEU-HOcs-his3+-λ-EU2, leu1-32, his3D1 (AW751)*
*h- smt0, urg1::RMCE_hphMX6_, LEU-HOcs-his3+-λ-EU2, rhp51::kanMX6 leu1-32, his3-D1 (AW686)*	pAW8E*Nde*I-HO-cyEGFP	*h- smt0, urg1::Purg1lox-HOcyEGFP, LEU-HOcs-his3+-λ-EU2, rhp51::kanMX6, leu1-32, his3D1 (AW734)*
	pAW8E*Nde*I-HO-cyEGFP-3XDSR	*h- smt0, urg1::Purg1lox-HOcyEGFP-3XDSR, LEU-HOcs-his3+-λ-EU2 rhp51::kanMX6, leu1-32, his3D1 (AW816)*
	pAW8E*Nde*I-HO-cyEGFP-4XDSR	*h- smt0, urg1::Purg1lox-HOcyEGFP-4XDSR, LEU-HOcs-his3+-λ-EU2,rhp51::kanMX6, leu1-32, his3D1 (AW818)*
	pAW8E*Nde*I-HO-cyEGFP-6XDSR	*h- smt0, urg1::Purg1lox-HOcyEGFP-6XDSR, LEU-HOcs-his3+-λ-EU2,rhp51::kanMX6, leu1-32, his3D1 (AW820)*
	pAW8E*Nde*I-HO-cyEGFP-8XDSR	*h- smt0, urg1::Purg1lox-HOcyEGFP-8XDSR, LEU-HOcs-his3+-λ-EU2, rhp51::kanMX6, leu1-32, his3D1 (AW822)*
	pAW8E*Nde*I-HO-cyEGFP-*spo5*DSR	*h- smt0, urg1::Purg1lox-HOcyEGFP-spo5DSR, LEU-HOcs-his3+-λ-EU2, rhp51::kanMX6, leu1-32, his3D1 (AW739)*
*h- smt0, urg1::RMCE_hphMX6_, RuiuR, rtf1::natMX6, leu1-32, nda3-KM311 (YSM077)*	pAW8E*Nde*I-rtf1-*spo5*DSR	*h- smt0, urg1::Purg1lox-rtf1-spo5DSR, RuiuR, rtf1::natMX6, leu1-32, nda3-KM311 (YSM098)*
*h+ urg1::RMCE_kanMX6_, leu1-32, ade6::ade6+-P_adh15_-skp1-AtTIR1-2NLS-9myc (AW617)*	pAW8E*Nde*I-*rhp18-HAIAA17*	*h+ urg1:: Purg1lox-rhp18-HAIAA17, leu1-32 ade6::ade6+-P_adh15_-skp1-atTIR1-2NLS-9myc, rhp18::kanMX6* * *(YDP210)*
	pAW8E*Nde*I-*rhp18-HAIAA17*-*spo5*DSR	*h+ urg1:: Purg1lox-rhp18-HAIAA17-spo5DSR, leu1-32 ade6::ade6+-P_adh15_-skp1-atTIR1-2NLS-9myc, rhp18::kanMX6* * *(YDP231)*
Other *S. pombe* strains used in the study		
	Strain number and source	Genotype
	*AW501 (Watson et al 2011)*	*h-, leu1-32*
	*JMM1015 (lab stock)*	*h- smt0, rhp51::kanMX6, ade6-704, leu1-32, ura4-D18*
	*AW507 (Watson et al 2011)*	*h-, urg1::Purg1lox-HO, LEU-HOcs-his3+-λ-EU2, his3-D1, leu1-32*
	*AW598 (lab stock)*	*h+, urg1::RMCE_kanMX6_, ade6-704, his3-D1, leu1-32*
	*YDP273 (this study)*	*h+, urg1::RMCE_kanMX6_, leu1-32, ade6::ade6+-P_adh15_-skp1-AtTIR1-2NLS-9myc, rhp18::natMX6*
	*HM2468 (Kanke et al., 2011)*	*h-, ade6::ade6+-P_adh15_-skp1-AtTIR1-2NLS-9myc*

* *= * following cassette exchange, *rhp18* ORF deleted with kanMX6 selectable marker using standard homologous recombination techniques

As discussed above, it is predicted that in HR deficient *rhp51*-delete cells the steady state level of *his3^+^* marker loss will increase compared to wild type cells because the alternative DSB repair pathway (HR) has been removed. The HO-cyEGFP Cre-expression plasmids were transformed into an *urg1* base strain containing the HOcs-HIS construct where the *rhp51* gene is also deleted (AW686 – Table 1). Following cassette exchange, the strains were again analysed in the P*_urg1lox_* OFF condition to determine the state-state level of histidine auxotrophic cells. For all the *rhp51-*delete strains studied, the rate of marker loss increased relative to the HR proficient WT strains (Figure 3B). The level increased in *P_urg1lox_-HO-cyEGFP-NO DSR*, *rhp51-d* cells to ∼ 43% compared to ∼ 11% in WT *rhp51* ^+^ cells. As observed for HR-proficient *rhp51* ^+^ cells, marker loss decreased in cells where HO expression was attenuated by DSR regulatory elements with the steady state levels in 8XDSR and spo5DSR cells around 2%.

We next preformed a time course to monitor HO-cyEGFP proteins levels following induction of P*_urg1lox_* by uracil addition. The samples were western blotted and probed with anti-GFP antibody (Figure 3C). As was observed for yEGFP protein levels (Figure 2D), following P*_urg1lox_* induction the kinetics of HO-cyEGFP protein increase was similar for *HO-cyEGFP-spo5DSR* cells and *HO-cyEGFP-NO DSR* cells, but a significant overall reduction in expressed protein levels was evident when the *spo5DSR* was present (Figure 3C). To investigate the kinetics *his3* ^+^ marker loss following induction of P*_urg1lox_*, cells containing either NO DSR, the *spo5DSR*, 3xDSR, 4xDSR, 6xDSR or 8xDSR were analysed for marker loss following induction by uracil (Figure 3D). The kinetics of marker loss was clearly influenced by DSR activity and correlated well with yEGFP levels observed in Figure 2. For example, in induced *P_urg1lox_-yEGFP*-*6xDSR* and *P_urg1lox_-yEGFP*-*spo5DSR* cells the protein levels were comparable (Figure 2A and 2B) and the levels and profile of *his3* ^+^ marker loss in *HO-cyEGFP-6XDSR* and *HO-cyEGFP-spo5DSR, HOcs-HIS* cells are also similar.

Overall, these data demonstrate that, despite being unable to detect the protein by western blot due to the low levels, the biological activity of the HO endonuclease in the P*_urg1lox_* OFF state is decreased by the presence of DSR motifs. This is consistent with protein levels in P*_urg1lox_* repressed cells being decreased when the transcript contains DSR elements. The implied “OFF” state protein levels mirror the protein levels observed by western blot analysis when the P*_urg1lox_* promoter was induced by uracil addition. Increasing the tandem core DSR repeat number showed increasing DSR activity, presumably reflecting RNA processing. Despite containing 6 DSR repeats, the 157bp *spo5* DSR element exhibits RNA processing activity in the “ON” state similar to that seen for 6 tandem core repeats (Figure 3B) but in the “OFF” state appears equivalent to 8XDSR repeats (see below). This suggests that other factors such as motif spacing may be important for efficient Mmi1 binding and RNA processing.

### Efficient regulation of replication fork barrier activity

In *S. pombe,* site-specific replication fork arrest and recombination-dependent fork restart have been studied extensively [15], [16], [17], [18]. The systems used involve the directional fork barrier sequence, *RTS1*, which is dependent for activity on the Myb-domain DNA binding protein Rtf1. To date, replication arrest at *RTS1* has been regulated by transcriptional control of the *rtf1* ^+^ gene via the thiamine repressible promoter, *nmt41*. However, the *nmt41* promoter is slow to induce (12–16 hrs) compared to the cell cycle time of *S. pombe* (2–3 hours). The *urg1* inducible system is quick to induce, with mRNA levels peaking 30 minutes after the addition of uracil [6]. However, previous attempts to regulate Rtf1 protein levels using *P_urg1lox_* were unsuccessful because the repressed level of P*_urg1lox_* transcription was too high for the system to be biologically off [7]. We therefore tested if the addition of the *spo5*DSR regulatory element was sufficient reduce Rtf1 “OFF” levels in P*_urg1lox_* repressed cells.

**Figure 4:**
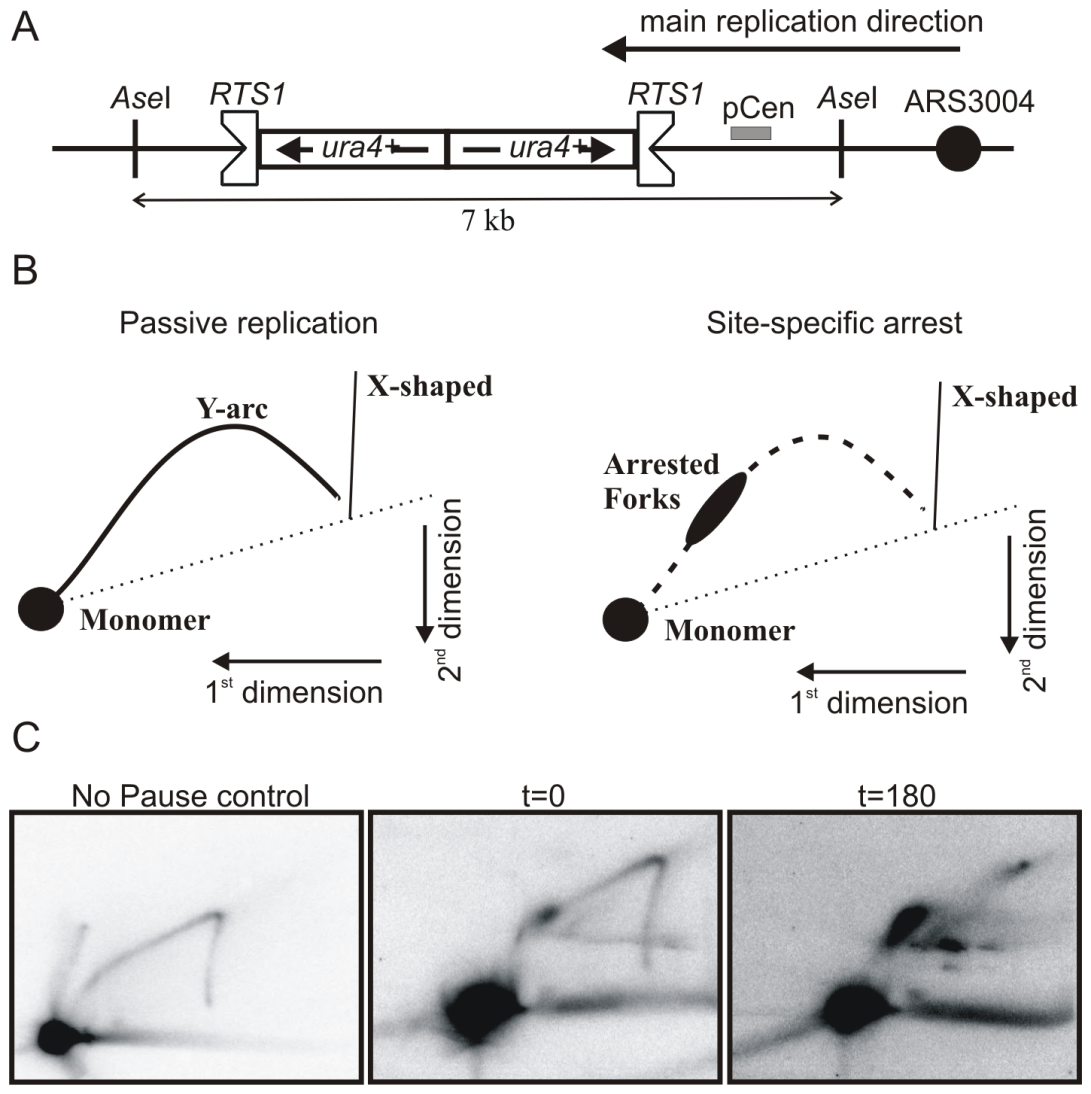
Use of the *S. pombe spo5* gene DSR element allows for tighter regulation of Rtf1 expression in an RTS1-dependent replication fork stall system. (A) Schematic illustration of inverted *ura4* repeat double RTS1 (*RuiuR*) construct. *RTS1* is a polar replication fork barrier. The triangular indent indicates the surface that prevents fork progression. (B) Cartoon representation of the expected replication intermediates (RIs) at the *RuiuR* locus as analysed by two-dimensional gel electrophoresis (2DGE). Left panel: RIs expected when the *Ase*I fragment indicated is replicated passively (no fork arrest at the *RTS1* barrier). Right panel - RIs expected in *RuiuR* cells upon fork arrest. (C) Left panel: control cells with no pause, demonstrating the position of the Y-arc. Middle and right panels: The *rtf1* ORF was inserted at the *urg1* locus in rtf1Δ cells by Cre-mediated cassette exchange to create YSM098 (see Table 1). The strain was grown in EMM+LA at 30°C (asynchronous culture) and Rtf1 protein induced by the addition of uracil at 0.25 mg/ml. Samples taken at time-points shown. Chromosomal DNA was digested by A*se*I, and RIs were analysed by 2DGE.

The study of template exchange following fork restart has involved a system in which two inverted copies of *ura4^+^* gene are flanked by *RTS1* sequences [17], [18]. This is referred to as the *RuiuR* construct (Figure 4A). We chose this system for testing the effectiveness of the *spo5* DSR element. The *RuiuR* construct was crossed into *urg1* base strain (AW469) to create YSM077 (Table 1). Using plasmid pAW8E*NdeI*-*rtf1-spo5*DSR (see materials and methods) we created YSM098 (*urg1::P_urg1lox_-rtf1-spo5DSR, RuiuR*) by Cre-mediated cassette exchange. Rtf1 activity in these cells can be monitored by detection of replication intermediates (RIs) arising from stalled replication forks using native two-dimensional gel electrophoresis (2DGE). Passive replication of the *RuiuR* locus (Rtf1 absent) is predicted to result in a Y-arc being detected (Figure 4B, left panel cartoon). However, upon site-specific fork arrest (Figure 4B, right panel cartoon), the intensity of the Y-arc is predicted to be reduced and an intense spot is predicted on the Y-arc, corresponding to the position of arrested forks.

In the complete absence of Rtf1 (*rtf1* deleted: *rtf1*D), replication this region is replicated passively (Figure 4C). When Rtf1 is under control of P*_urg1lox_* in association with the s*po5*DSR and repressed (Figure 4C; t = 0), the Y-arc is clearly visible with a faint spot corresponding to a low level of replication fork stalling. This is presumably because, as seen for the regulation HO using DSR sequences, the DSR/Mmi1 pathway of mRNA degradation is not 100% efficient. 180 minutes after the addition of uracil (Rtf1 induced), the Y-arc is no longer visible and an intense spot arising from fork arrest is seen (Figure 1C; t = 180). These data show that the degradation of the *rtf1-spo5DSR* mRNA reduces the cellular concentrations of Rtf1 protein sufficiently to allow use of the rapidly inducible P*_urg1lox_* system to study blocked replication forks by 2DGE. Importantly, this will allow the study of synchronised cells cultures to further elucidate the mechanisms of recombination-dependent fork restart in *S. pombe*.

### Production of a null rhp18 phenotype by the addition of an auxin-inducible protein depletion system

**Figure 5:**
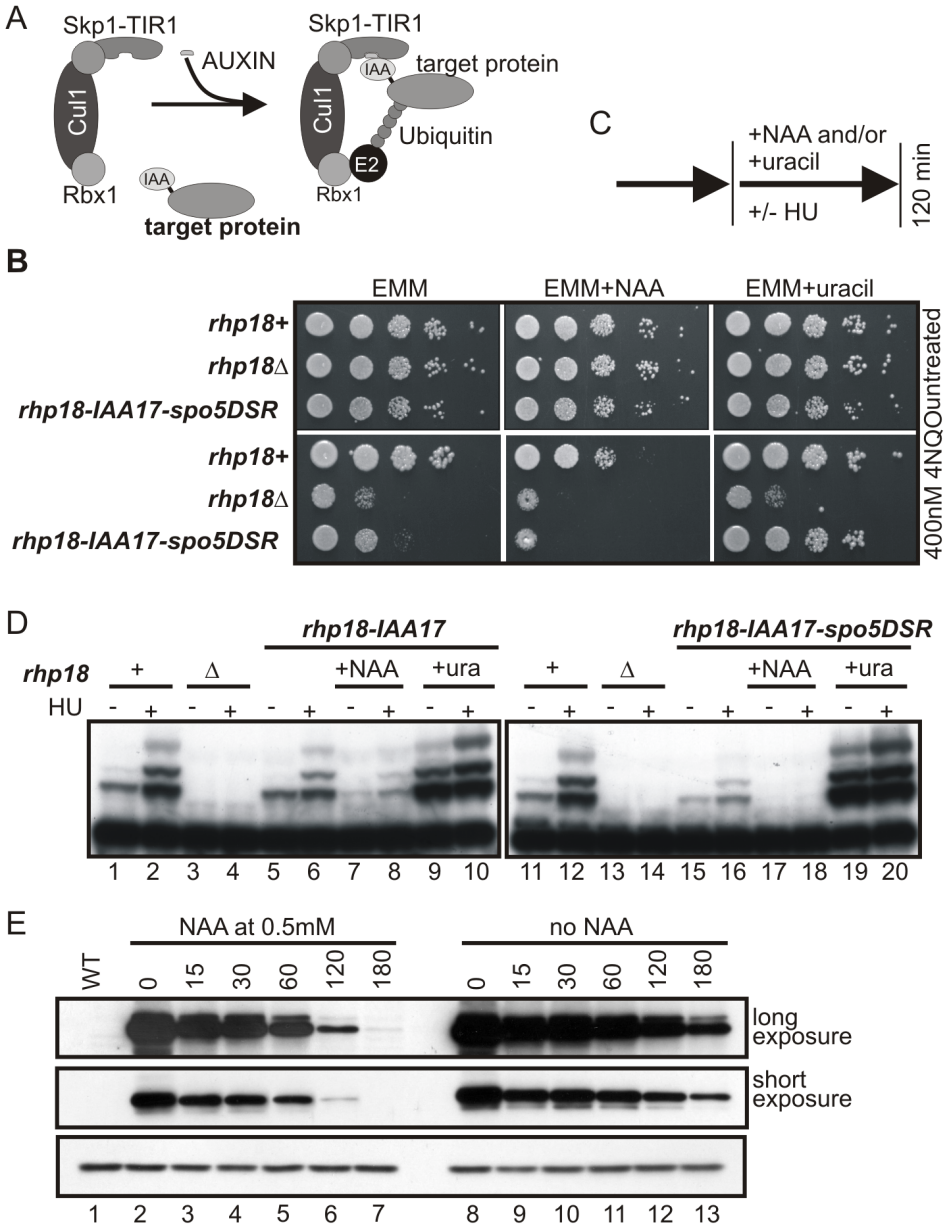
Use of an auxin-inducible degron allows for the generation of a conditional *rhp18* mutant strain. (**A**) Cartoon of the IAA17 degron system: addition of auxin allows binding of the TIR adaptor (fused to Skp1) to the IAA17 tag (IAA), which is fused to the target protein. This induces ubquitination and proteasome degradation. (**B**) Strains AW617 (*rhp18* ^+^), YDP273 (*rhp18*Δ) and YDP231 (*rhp18*Δ, *P_urg1lox_-rhp18-HAIAA17-spo5DSR*) were serially diluted 10-fold in water and spotted on EMM+L plates supplemented as shown with uracil at 0.25 mg/ml, NAA at 0.5 mM and/or 4NQO at 400 nM. Time of incubation at 30°C: Top panels 3 days, bottom panels 5 days. (**C**) Schematic of experimental procedure used in D. HU  =  hydroxyurea, NAA  =  1-naphthaleneacetic acid. (**D**) Ubiquitination of PCNA is abolished in *P_urg1lox_-rhp18-HAIAA7-spo5DSR* cells in the presence of NAA. Logarithmically growing AW617 (*rhp18* ^+^), YDP273 (*rhp18-*delete), YDP210 (*rhp18-*delete, *P_urg1lox_-rhp18-HAIAA17*) and YDP231 (*rhp18-*delete, *P_urg1lox_-rhp18-HAIAA17-spo5DSR*) cells cultured in EMM+L at 30°C untreated (−) or treated with 10 mM HU (+) and grown for a 120 minutes or grown for 120 minutes in the presence of NAA at 0.5 mM or uracil at 0.25 mg/ml. Total protein extracts were separated by SDS PAGE prior to Western blotting using anti-PCNA antibody. (**E**). The auxin degron promotes protein degradation upon “shut-off”. P*_urg1/lox_*. YDP210 (*rhp18-*delete, *P_urg1lox_-rhp18-HAIAA17*) cells were grown in EMM+L and *P_urg1lox_* induced by the addition of uracil at 0.25 mg/ml. After 3 h induction, cells were pelleted by centrifugation, washed twice in EMM+L and re-suspended in EMM+L. Samples taken at time-points shown (mins). Total protein extracts were separated by SDS PAGE prior to Western blotting revealed protein levels using anti-HA to detect Rhp18-HAIAA17 (upper panels) and anti-tubulin to detect tubulin as a loading control (lower panel). WT represents control strain AW501 (*h* ^−^, *leu1-32*).

The above experiments demonstrate that the addition of DSR sequences to destabilise the transcripts produced by the basal level of uninduced P*_urg1lox_* provides a level of attenuation of the “OFF” level of protein function that is sufficient to allow the manipulation of a cellular function that is sensitive to low levels of protein. However, additional control of the protein stability would offer two additional advantages: first, it would allow even greater control of “OFF” level function and second, it would allow more rapid removal of residual protein upon “shut off” of P*_urg1_* transcription, which would add to the versatility of the system. To establish a test system to validate the utility of combining the auxin degron (Figure 5A) with our P*_urg1lox_* DSR system, we turned to a well characterised DNA repair function; Rhp18-dependent post replication repair (PRR). Rhp18 is the homolog of *S. cerevisiae* Rad18. The *S. pombe* Rhp18^Rad18^ ubiquitin ligase is essential for PRR, allowing cells to progress through and survive S-phase in the presence of replication blocking lesions [19]. *rhp18^rad18^* delete mutants are hypersensitive to DNA-damaging agents [20], allowing us to test for a null allele phenotype. Together with Rhp6 (*S. cerevisiae* Rad6 homologue), Rhp18^Rad18^ mono-ubiquitylates the sliding clamp protein proliferating cell nuclear antigen (PCNA; Pcn1 in *S. pombe*) in S phase and in response to DNA lesions [19], [21].

Using RMCE, we created strains where the *rhp18^rad18^* ORF, tagged at the C-terminus with the IAA17 degron, was inserted at the *urg1* locus either with or without the *spo5*DSR element. Plasmids pAW8E*NdeI-rhp18IAA17* and pAW8E*NdeI-rhp18IAA17spoDSR* (see materials and methods) and the *urg1* base strain YDP273 were used to create strains YDP210 (*P_urg1lox_-rhp18-cIAA17*) and YDP231 (*P_urg1lox_-rhp18-cIAA17-spo5DSR*) respectively (Table 1). Base strain YDP273 also contains the *P_adh15_-skp1-AtTIR1* fusion necessary for the efficient poly-ubiquitination of the IAA17 degron tag (Kanke et al 2011). Strains YDP210 (*rhp18^rad18^-delete, P_urg1lox_-rhp18-cIAA17-spo5DSR,*), AW617 (*rhp18^rad18+^*) and YDP273 (*rhp18^rad18^*-delete) were serially diluted and spotted onto YEA media (control) and YEA media containing the UV memetic 4-Nitro-Quinoline-1-Oxide (4NQO) (Figure 5B). To regulate Rhp18^Rad18^ induction, uracil was added or omitted from the growth media. To regulate Rhp18^Rad18^ stability, the synthetic plant auxin NAA was either added or omitted. Following growth at 30°C, a null *rhp18^rad18^* phenotype was only observed in *rhp18-cIAA17-spo5DSR* cells where P*_urg1lox_* expression is repressed (uracil absent) and auxin-dependent Rhp18-IAA7 degradation induced (NAA present) (Figure 5B bottom middle panel). The *rhp18-cIAA17-spo5DSR* cells were only partially sensitive to 4NQO in the absence of NAA (Figure 5B bottom left panel) demonstrating that transcription repression and RNA processing alone are insufficient to obtain the desired phenotype. These results demonstrate that protein destabilisation can add a further level of control when proteins are regulated via transcription from P*_urg1lox_*.

Rhp18^Rad18^ is required for the ubiquitination of Pcn1 (*S. pombe* PCNA homolog), which occurs during S-phase and accumulates in cells treated with hydroxyurea [19]. Thus, the levels of Ub-Pcn1 in growing and hydroxyurea-treated cells provides a biochemical readout of Rhp18^Rad18^ activity. To compare the utility of the auxin degron, the regulation by DSR motifs and the combination of the two together we thus explored the levels of Ub-Pcn1 in a variety of strains and conditions. Cells in which Rhp18^Rad18^-IAA17 is regulated by P*_urg1lox_* either with or without an associated *spo5*DSR element were grown to mid-log phase and either treated, or not, with 10 mM hydroxyurea. Where appropriate, 0.25 mg/ml of uracil was added to induce Rhp18^Rad18^-IAA17 and 0.5 mM NAA was added to induce Rhp18^Rad18^-IAA17 instability (for a schematic of experimental design, see Figure 5C). After 120 minutes incubation at 30°C, cell extracts were prepared and analysed by western blot using an α-PCNA antibody (Figure 4D). As expected, in the control *rhp18^rad18+^* cells (*rhp18*+), higher molecular weight Ub-Pcn1 species were observed in both logarithmically growing cells and, at higher levels, hydroxyurea arrested cells (Figure 5D, lanes 1 and 2; 11 and 12). These modifications were absent in the *rhp18^rad18^*-deleted control (*rhp18*Δ) strain (Figure 5D, lanes 3 and 4; 13 and 14).

In both untreated and hydroxyurea-treated *rhp18^rad18^-cIAA17* (YPD210) and *rhp18^rad18^-cIAA17-spo5DSR* (YDP231) cells, the levels of Ub-Pcn1 decreased in the repressed conditions (uracil absent) when compared to *rhp18^rad18+^* (Figure 5D, lanes 5 and 6; 15 and 16). However, significant residual signal remained, even in the DSR-containing construct. Thus, while repressed P*_urg1lox_* transcript levels appear lower that of the endogenous *rhp18^rad18^* locus and this is further reduced by the presence of the *spo5*DSR, biological function is not completely ablated. When cells were concomitantly treated with the synthetic auxin, NAA, modification levels were further decreased in both strains. Importantly, Ub-Pcn1 was undetectable in both untreated and HU-treated *rhp18-cIAA17-spo5DSR* cells (Figure 5D, lanes 17 and 18), while residual levels of modifications remained in *rhp18-cIAA17* cells (Figure 5D, lanes 7 and 8). Over-expression of *P_urg1lox_-rhp18-IAA17* (presence of uracil, absence of auxin) results in higher levels of PCNA ubiquitilation when compared to control *rhp18^rad18+^* cells (Figure 5D, lanes 9 and 10; 19 and 20).

To establish if, upon shut-off of P_urg1lox_-dependent transcription, auxin addition resulted in more rapid removal of CIAA17-tagged protein, we grew cells in the presence of uracil for 3 hours before transferring them to fresh media without uracil, either supplemented, or not, with auxin (Figure 5E). Loss of the GFP signal was more rapid in the presence of auxin. Taken together, these results show that control over transcription rate, RNA turnover and protein depletion may all be required to obtain a null allele phenotype.

### Influence of arginine and urea

**Figure 6:**
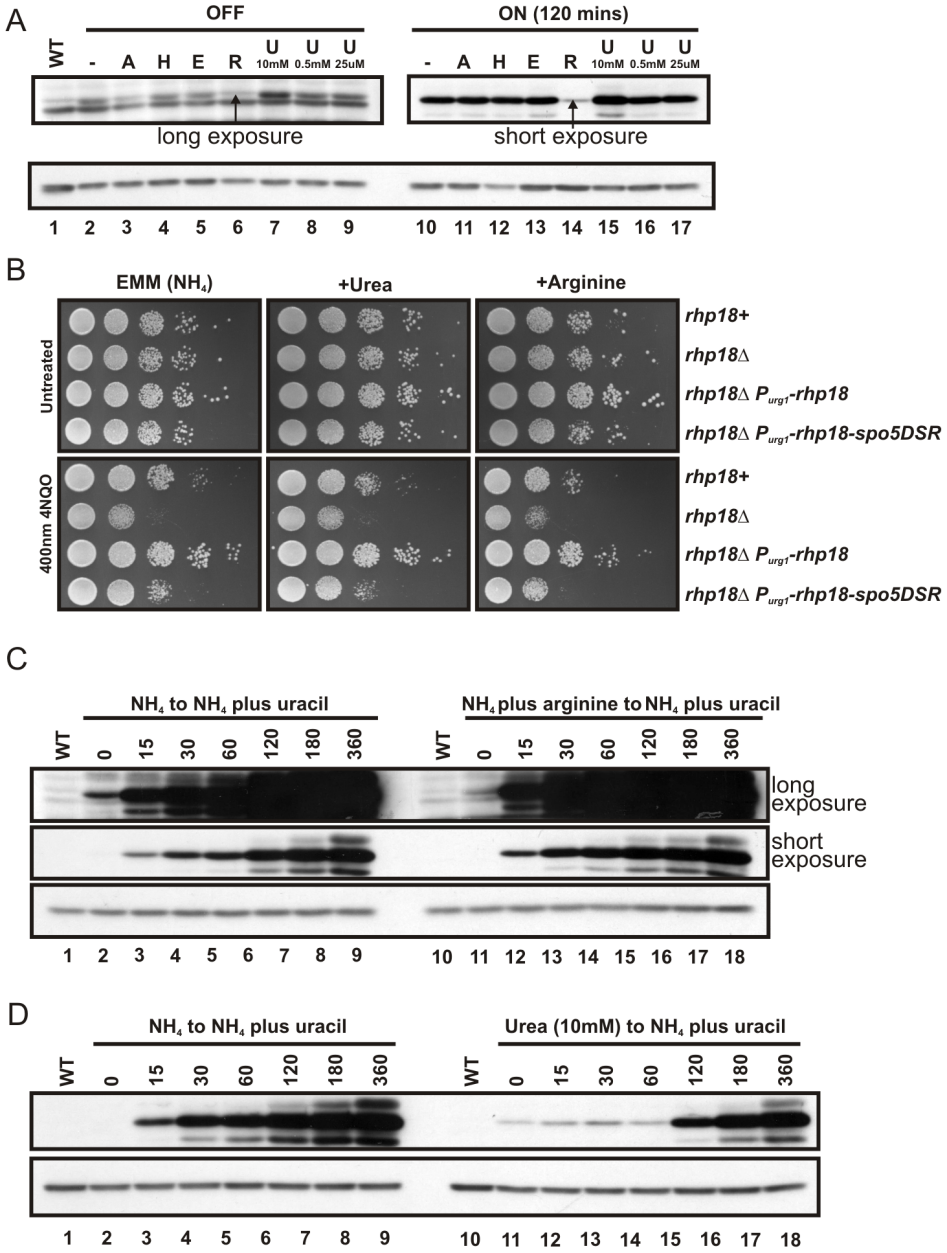
The effect of arginine and urea on P*_urg1lox_* expression levels. (**A**) Arginine reduces both *P_urg1lox_* induced and repressed protein levels. AW640 (*P_urg1lox_-cyEGFP*) cells were grown in EMM+L or EMM+L supplemented with adenine (A), histidine (H), arginine (R), EMM media where NH_4_Cl was substituted for 22 mM glutamic acid (E), EMM media where NH_4_Cl was substituted for urea at 10 mM (U 10 mM) or EMM media supplemented with urea at 0.5 mM (U 0.5 mM) or 25 µM (U 25 µM). Cells were induced by the addition of uracil at 0.25 mg/ml and cells grown for 2h. A long exposure of the P*_urg1lox_* OFF and a short exposure of the P*_urg1lox_* ON are shown. Arrows mark band of interest. (**B**). Strains AW617 (*rhp18* ^+^), YDP273 (*rhp18*Δ), YDP210 (*rhp18*Δ, *P_urg1lox_-rhp18*) and YDP231 (*rhp18*Δ, *P_urg1lox_-rhp18-spo5DSR*) were serially diluted 10-fold in water and spotted on EMM+L plates or supplemented as shown with urea at 25 uM or arginine at 100 µg/ml, with or without 4NQO at 400 nM. Time of incubation at 30°C was 3 days. (**C**). The induction kinetics of *P_urg1lox_* is improved when cells are pre-cultured in EMM supplemented with arginine. AW640 (*P_urg1lox_-cyEGFP*) cells were pre-cultured in EMM+L or EMM+L supplemented with arginine at 100 µg/ml (P*_urg1lox_* OFF). Cells were pelleted by centrifugation, washed twice in EMM+L and re-suspended in EMM+L containing uracil at 0.25 mg/ml (P*_urg1lox_* ON). Samples taken at time-points shown (mins). (**D**). The induction kinetics of *P_urg1lox_* is significantly delayed when cells are pre-cultured in the presence of urea. AW640 (*P_urg1lox_-cyEGFP*) cells were pre-cultured in EMM+L or EMM+L where the nitrogen source is 10 mM urea (P*_urg1lox_* OFF). Cells were pelleted by centrifugation, washed twice in EMM+L and re-suspended in EMM+L containing uracil at 0.25 mg/ml (P*_urg1lox_* ON). Samples taken at time points shown (mins). For (A), (C) and (D), total protein extracts were separated by SDS PAGE prior to Western blotting using anti-GFP to detect yEGFP (upper panels) and anti-tubulin to detect tubulin as a loading control (lower panel). WT represents control strain AW501 (*h* ^−^, *leu1-32*).

During the course of our experiments, we have noticed that the level of P*_urg1lox_*-dependent transcription was significantly reduced in cells grown in EMM media containing arginine. Subsequent experiments demonstrated that, uniquely amongst the commonly used amino acid supplements, arginine significantly suppress the “ON” level (uracil present) of P*_urg1lox_*-dependent GFP expression (Figure 6A, lane 14). Importantly, the “OFF” level (uracil absent) was also reduced when compared to cells pre-cultured in the absence of arginine (Figure 6A, lane 6 versus lane 2) (see also figure 6C, lane 11 versus lane 2). A similar reduction was seen with the presence of adenine in this experiment, but unlike that seen with arginine, this was not always reproducible. To further improve the P*_urg1lox_* system, we thus investigated the potential use of arginine for reducing “OFF” level transcription. We first tested if, biologically, the presence of arginine could increase the sensitivity observed when *rhp18* is under the control of P*_urg1lox_* and is suppressed by the absence of uracil (Figure 6B). Indeed, when grown in the absence of uracil (no induction) and the presence of arginine (inhibition), the phenotype of *rhp18-d*, P*_urg1lox_*-*rhp18-spo5DSR* cells was closer to that seen for the *rhp18* null mutant. We next investigated the kinetics of induction for P*_urg1lox_* in cells pre-cultured in arginine-containing medium and transferred into fresh arginine-free medium and induced immediately by addition of uracil (Figure 6D). To our surprise, the kinetics of induction was improved, with higher levels of yEGFP present at the earlier time points when compared to cells pre-cultured without arginine. Levels of yEGFP were comparable 3 hours post induction (Figure 6C). The use of arginine in the pre-culture can therefore markedly increase the dynamic range of the P_urg1lox_ promoter system and increase the speed of induction.

A novel uracil catabolic pathway has recently been described in the budding yeast *Saccharomyces kluyveri*. This pathway is dependent on a pair of genes, *URC1* and *URC4*, that are highly conserved in many bacteria and fungi [22]. The *S. kluyveri URC1* and *URC4* genes are the orthologs of the *S. pombe urg1^+^* and *urg3^+^*, respectively. In *S. kluyveri*, Urc1, together with Urc4 and a set of other enzymes, breaks down uracil into urea and 3-hydroxypropionic acid [22]. When considering that the early commitment step of a metabolic pathway is usually subject to feedback inhibition by the final product of that pathway [23] and the fact that arginine can be broken down into urea by arginase when nitrogen is limiting [24] **,** it is conceivable that Urc1/Urg1 expression or activity might be subject to negative regulation by urea. As *S. pombe* can use urea as a sole nitrogen source, we explored if replacing the ammonium in the EMM growth media with urea would have a similar effect on expression levels as was observed for arginine. Compared to cells grown in ammonim-containing media, there was no significant decrease of the “ON” level of GFP when urea was used as the sole nitrogen source (Figure 6A, lane 15), indeed the level was higher (lane 15 versus lane 2). The equivalent “OFF” level was also higher (Figure 6A, lane 7). Furthermore, no reduction in the “ON” level of P*_urg1lox_*-dependent GFP expression was seen in ammonium-EMM media supplemented with lower concentrations of urea (0.5 mM and 25 uM) (Figure 6A lanes 16 and 17). As seen in Figure 6B, the addition of 25 uM urea also had no effect on the sensitivity of *P_urg1lox_-rhp18* cells to 4NQO. Despite these observations, an initial inhibition of yEGFP induction was evident when cells were grown in the presence of 10 mM urea before being transferred into fresh urea-free medium (with ammonium as a nitrogen source) and induced by addition of uracil (Figure 6D). Thus, while urea does directly or indirectly have an effect on *urg1* promoter activity, a simple model of substrate inhibition does not explain the complexity of P*_urg1_* regulation.

## Discussion

For *S. pombe*, the control of gene expression has remained a problem for many years because a rapidly and easily inducible transcriptional regulation system, i.e. one equivalent to the P*_GAL_* system of *S. cerevisiae*, has not been available. A number of regulatable expression systems have been established characterised, and each has advantages and disadvantages. The *nmt1* promoter has most commonly been used to manipulate protein levels, and thus gene function, because it presents several distinct advantages: first, it is functional when integrated at different sites in the genome; second, it has a good dynamic range (∼75 fold in our hands); third, through the use of TATA-box mutations several different strengths of promoter are available. Importantly, these maintain the dynamic range between “ON” and “OFF” states. However, the *nmt1* promoter has one major disadvantage: it takes between 12 and 16 hours to induce and induction is not particularly synchronous. This has limited its utility for the many experiments that require rapid and synchronous induction to study, for example, the cell cycle specificity of a proteins function.

The recently described *urg1* ^+^ promoter [6], which is induced by the addition of uracil to the media, offers the most plausible alternative to P*_nmt1_* since it has a similar dynamic range (∼1:75) and is induced within 30 minutes by a simple media manipulation: the addition of uracil, which does not otherwise significantly alter cell physiology. However, P*_urg1_* does suffer from a number of disadvantages: first it does not work well outside of its normal locus; second, its basal level of transcription is relatively high; and third, it is induced during meiosis. In previous work [7] we described a system that overcame the first of these disadvantages. We established a Recombination Mediated Cassette Exchange system that allowed the rapid and simple replacement of the *urg1* ORF with any sequence of interest. In this report, we have overcome the second of these disadvantages by providing two additional levels of regulation: one at the level of RNA stability and the second at the level of protein stability.

While we do not provide any analysis of how the P*_urg1_* promoter functions, we note two things that may be informative: First, the locus is part of a widely conserved operon that has been shown to carry out a novel uracil catabolic cascade in response to nitrogen availability. In addition to the *urg1^+^* and *urg3^+^* genes, this operon also includes genes that are predicted to encode for a uracil transporter, uracil phosphoribosyltransferases, and perhaps most importantly, a putative transcription factor, which belongs to the Zinc finger family of transcription factors [22]. The conserved genomic organization of this bouquet of genes in a wide diversity of fungi and bacteria suggests that the transcriptional regulation of *urg1^+^* expression is likely to be complex. This may be one reason why it has not been possible to transfer the dynamic range of the P*_urg1lox_* promoter available at the native locus to a plasmid-based system. Second, we observed that arginine, when supplemented into the growth media, represses uracil-dependent induction by P*_urg1/lox_*. In particular, arginine suppresses the “OFF” state transcription – i.e. reducing the “leakiness” of the promoter and improves the induction kinetics. Our analysis shows that, serendipitously, this provide an additional opportunity, when combined with our DSR sequences and/or cIAA17 degron tag, to tightly regulate processes that are particularly sensitive to very low protein levels.

While the use of the modified P*_urg1_* system we describe here will not solve all the problems associated with gene regulation in *S. pombe*, we have demonstrated, both here and in our own unpublished data, that the system is both versatile, robust, easy to use and applicable to a range of biological questions. Most importantly, we have succeeded in regulating protein functions which are sensitive to low levels of protein in cells and have exploited the system to study induced conditions in a cell cycle-dependent manner. For our own purposes we chose to regulate HO-dependent site-specific DSB formation, Rtf1-dependent replication fork arrest and Rhp18^Rad18^-dependent post replication repair. However, other functions can also be regulated by application of this modified P*_urg1lox_* system. The availability of the generic “base strain” required for RMCE and the convenience of a range of plasmids compatible with the Cre-mediated site specific recombination on which RMCE is based, mean that any sequence can be simply and easily cloned into an appropriate RMCE plasmid and targeted directly to the *urg1* locus via a simple transformation and selection procedure at an efficiency that is routinely greater than 50% of cells. Once integrated, the sequence (usually an ORF) will be under transcriptional control such that it can be regulated simply by the addition of uracil. Based on which RMCE vector the sequence of interest is cloned into, both the “ON” and “OFF” state transcript levels can be attenuated by the desired amount due to the inclusion of one or more DSR elements in the non-translated region of the resulting transcript. Similarly, protein stability can be regulated by inclusion of a protein tag derived from the Arabidopsis IAA17 degron, which is regulated by the addition of auxin. Here we have shown that both these systems function and that they can be combined to generate genuine conditional null allele phenotypes. Finally, the vercitility of the *P_urg1lox_* system can be further enhanced by the simple addition of arginine in pre-induction cultures.

## Materials and Methods

### Strains and growth conditions

Strains used in this work are listed in Table 1 and all strains grown at 30°C. The media composition was as described [25]. The nitrogen source used in Edinburgh Minimal Media was either 5 g/litre NH_4_Cl (94 mM), 3.75 g/litre L-glutamic acid (22 mM) or 0.6 g/litre urea (10 mM). In the text, EMM refers to nitrogen source used as NH_4_Cl unless stated otherwise. For selection of G418, hygromycin (HPH) and nourseothricin (NAT) resistant cells, G418 disulsuphate (Melford), hygromycin B (Melford) and nourseothricin-dihydrogen sulphate (Melford) were added to YEA plates at a final concentration of 200 µg/ml, 200 µg/ml and 100 µg/ml respectively. Synthetic plant auxin 1-Naphthaleneacetic acid (NAA) (Sigma) powder was dissolved in a small volume of 0.1N NaOH and then diluted with double distilled water to the required concentration (0.5 M). EMM media was supplemented with leucine (L), adenine (A), arginine (R) and histidine (H) at 100 µg/ml as required. Yeast transformations were performed using a lithium acetate method [26]. Cell pre-cultures for P*_urg1lox_* induction assays were not grown to stationary phase before sub-culturing. *E. coli* strain DH5α was used for all cloning procedures.

### Construction of DSR plasmids for cassette exchange at the *urg1* locus

Complimentary oligonucleotides containing 1 to 8 repeats of the DSR core element (TTAAAC) (1xDSR to 8xDSR) and 8 repeats of the mutated core element (GTAAAC) (8mxDSR) were synthesised (P1 to P18 - Table 2). The core motifs were separated by 6 nucleotides of randomly selected bacteriophage lambda DNA sequence. After annealing complimentary oligonucleotides, the resulting DNA duplex was flanked by overhangs compatible with *Bgl*II and *Xma*I restriction enzymes. The annealed oligonucleotides were ligated into *Bgl*II/*Xma*I restricted pAW8E*Nde*I-CTAP [7], replacing the CTAP tag to create pAW8E*Nde*I-L-1xDSR through to pAW8E*Nde*I-L-8xDSR and pAW8E*Nde*I-L-8mxDSR. The yeast codon optimised yEGFP ORF from pAW8E*Nde*I-cyEGFP (Watson et al 2011) was sub-cloned as a *Bgl*II fragment into the DSR plasmids to create pAW8E*Nde*I-cyEGFP-1xDSR through to pAW8E*Nde*I-cyEGFP-8xDSR and pAW8E*Nde*I-cyEGFP-8mxDSR. The 3HA sequence (encoding 3 copies of the hemagglutinin epitope tag) from pAW8E*Nde*I-c3HA (Watson et al., 2011) was sub-cloned as a *Bgl*II fragment into pAW8E*Nde*I-L-3xDSR, pAW8E*Nde*I-L-4xDSR, pAW8E*Nde*I-L-6xDSR and pAW8E*Nde*I-L-8xDSR to create pAW8E*Nde*I-c3HA-3xDSR, pAW8E*Nde*I-c3HA-4xDSR, pAW8E*Nde*I-c3HA-6xDSR and pAW8E*Nde*I-c3HA-8xDSR respectively.

**Table 2:** Primers used in this study.

NAME	SEQUENCE (5' TO 3')
P1	GATCTTTAAACC
P2	CCGGGGTTTAAA
P3	GATCTTTAAACTCCGTATTAAACC
P4	CCGGGGTTTAATACGGAGTTTAAA
P5	GATCTTTAAACTCCGTATTAAACCCATTCTTAAACC
P6	CCGGGGTTTAAGAATGGGTTTAATACGGAGTTTAAA
P7	GATCTTTAAACTCCGTATTAAACCCATTCTTAAACAGAACTTTAAACC
P8	CCGGGGTTTAAAGTTCTGTTTAAGAATGGGTTTAATACGGAGTTTAAA
P9	GATCTTTAAACTCCGTATTAAACCCATTCTTAAACAGAACTTTAAACGGCAGGTTAAACC
P10	CCGGGGTTTAACCTGCCGTTTAAAGTTCTGTTTAAGAATGGGTTTAATACGGAGTTTAAA
P11	GATCTTTAAACTCCGTATTAAACCCATTCTTAAACAGAACTTTAAACGGCAGGTTAAACGTAATGTTAAACC
P12	CCGGGGTTTAACATTACGTTTAACCTGCCGTTTAAAGTTCTGTTTAAGAATGGGTTTAATACGGAGTTTAAA
P13	GATCTTTAAACTCCGTATTAAACCCATTCTTAAACAGAACTTTAAACGGCAGGTTAAACGTAATGTTAAACAGGTGCTTAAACC
P14	CCGGGGTTTAAGCACCTGTTTAACATTACGTTTAACCTGCCGTTTAAAGTTCTGTTTAAGAATGGGTTTAATACGGAGTTTAAA
P15	GATCTTTAAACTCCGTATTAAACCCATTCTTAAACAGAACTTTAAACGGCAGGTTAAACGTAATGTTAAACAGGTGCTTAAACTTTATGTTAAACC
P16	CCGGGGTTTAACATAAAGTTTAAGCACCTGTTTAACATTACGTTTAACCTGCCGTTTAAAGTTCTGTTTAAGAATGGGTTTAATACGGAGTTTAAA
P17	GATCTGTAAACTCCGTAGTAAACCCATTCGTAAACAGAACTGTAAACGGCAGGGTAAACGTAATGGTAAACAGGTGCGTAAACTTTATGGTAAACC
P18	CCGGGGTTTACCATAAAGTTTACGCACCTGTTTACCATTACGTTTACCCTGCCGTTTACAGTTCTGTTTACGAATGGGTTTACTACGGAGTTTACA
P19	AAAACCCGGGACTACGCCATATCATGCCCA
P20	AAAACCCGGGGCTTTGTCTAACAGGTTTTATGTTGGTTTAAGT
P21	AAAAAGATCTATGATGGGCAGTGTCGAGCT
P22	AAAACCCGGGTCAAGCTCTGCTCTTGCACTTCTC
P23	CTAGTGGTTATCCTTATGATGTTCCTGATTATGCTT
P24	CTAGAAGCATAATCAGGAACATCATAAGGATAACCA
P25	CCCCATATGCAAGGAAAAAACAATTTAAGTTGCAGA
P26	CCCACTAGTGCATAAATCATCGGCGTTAGAAAAAGC
P27	GCGAGAGACCTTCTTATTAAAACCAAAAGACTTCC
P28	ATAAGAAGGTCTCTCGCAGCCACA
P29	AAAACATATGCAAGGAAAAAACAATTTAAGTTGCAGACC
P30	AAAAAGATCTCTAGCATAAATCATCGGCGTTAGAAAAAGC
P31	TTTAAATCAAATCTTCCATGCG
P32	GATGCCAGACCGTAATGACAAAA

The 157bp DSR element of the *S. pombe spo5* gene as identified by Harigaya et al. (2006) was amplified using the KOD HotStart DNA polymerase system (Novagen - used for all subsequent PCR reactions) from total genomic DNA using primers P19 and P20 (Table 2). The product was cloned into *Xma*I restricted pAW8E*Nde*I-cyEGFP to create pAW8E*Nde*I-cyEGFP-*spo5*DSR. The 3HA sequence from pAW8E*Nde*I-c3HA was sub-cloned into pAW8E*Nde*I-cyEGFP-*spo5*DSR as *Bgl*II fragment to create pAW8E*Nde*I-c3HA-*spo5*DSR.

The IAA17 degron tag sequence was amplified from the plasmid template pMK43 [13] using primers P21 and P22 and the resulting fragment was cloned into the *Bgl*II site of pAW8E*Nde*I-cyEGFP and pAW8E*Nde*I-cyEGFP-*spo5*DSR, replacing the yEGFP sequence, to create pAW8E*Nde*I-cIAA17 and pAW8E*Nde*I-cIAA17-*spo5*DSR respectively. A single copy of the HA hemagglutinin epitope tag was inserted between the MCS and the poly-TGS linker by annealing complimentary oligonucleotides P23 and P24 and cloning the resulting DNA duplex into *Spe*I restricted pAW8E*Nde*I-cIAA17 and pAW8E*Nde*I-cIAA17-*spo5*DSR to create pAW8E*Nde*I-cHAIAA17 and pAW8E*Nde*I-cHAIAA17-*spo5*DSR respectively.

To create non-tagging Cre-expression DSR plasmids, the sequence located between the loxP and loxM3 sites of pAW8E*Nde*I (Figure 1) was replaced with a construct containing the 37bp *urg1* promoter fragment, an MCS of *Nde*I-*Sph*I-*Sac*I-*Sal*I-*Spe*I and the required DSR sequence. Constructs were synthesised (Genscript) and sub-cloned into *Nhe*I/*Xma*I restricted pAW8E*Nde*I to create pAW8E*Nde*I-3xDSR, pAW8E*Nde*I-4xDSR, pAW8E*Nde*I-6xDSR, pAW8E*Nde*I-8xDSR and pAW8E*Nde*I-*spo5*DSR.

Plasmids created are listed in Figure 1 including Genbank accession numbers for each. Plasmids are available from Addgene.

### DSR plasmid inserts

The *S. pombe rtf1* ORF was amplified from total genomic DNA using primers P25 and P26 and cloned into pAW8E*Nde*I-cyEGFP as an *Nde*I/*Spe*I fragment to generate pAW8E*Nde*I-*rtf1*-cyEGFP. The *Bgl*II restriction enzyme site was removed from the *rtf1* ORF of pAW8E*Nde*I-*rtf1*-cyEGFP using the QuikChange Site-Directed Mutagenesis Kit (Stratagene) and the primers P27 and P28. The mutated *rft1* ORF was amplified using P29 and P30 and cloned into pAW8E*Nde*I-cyEGFP*spo5*DSR as an *Nde*I/BglII fragment, removing cyEGFP tag, to generate pAW8E*Nde*I-*rtf1*-*spo5*DSR. The *rtf1* plasmid insert was confirmed by sequencing.

The HO endonuclease ORF was sub-cloned from pAWE*Nde*I-HO-cyEGFP [7] as an *Nde*I/*Spe*I fragment into pAW8E*Nde*I-cyEGFP-DSR plasmids to create pAWE*Nde*I-HO-cyEGFP, pAWE*Nde*I-HO-cyEGFP-3xDSR, pAWE*Nde*I-HO-cyEGFP-4xDSR, pAWE*Nde*I-HO-cyEGFP-6xDSR, pAWE*Nde*I-HO-cyEGFP-8xDSR, and pAWE*Nde*I-HO-cyEGFP-*spo5*DSR.

### *S. pombe* strain construction

All P*_urg1lox_* strains were generated using Cre-mediated cassette exchange. See Table 1 for a list of strains created, plus the base strain and pAW8E*Nde*I Cre-expression plasmid used for each. Other strains used in this study are also listed in Table 1. To create *urg1* base strain AW686, strains AW469 and JMM1015 were crossed (Table 1). The *urg1* base strain AW617 was generated by crossing HM2468 with AW598 (Table 1). The *rhp18^rad18^* gene locus in AW617 was deleted using the *natMX6* selectable marker to create strain YDP273 (Table 1).

### Cassette exchange

Cassette exchange was performed essentially as described [7]. The procedure was adapted for the introduction of HO-endonuclease gene sequences into HR deficient *rhp51*-delete *urg1* base strains containing the HOcs single strand annealing (SSA) system. After transformation of the Cre-expression plasmids containing the HO gene into the *rhp51*-delete *urg1* base strain AW686 (Table 1), cells were plated directly onto EMM plates supplemented with 15 µM thiamine (EMM+T - P*_nmt1_* OFF). Following incubation at 30°C for 4–5days, transformants were re-streaked onto fresh EMM+T plates. Transformants were grown in 50 mls liquid EMM media supplemented with leucine but with thiamine omitted (EMM+L) overnight to approximately 1×10^6^ cells/ml and 500 cells plated onto EMM+L plates and grown at 30°C until colonies appear. Colonies were replica plated onto YEA plates supplemented with hygromycin at 200 µg/ml. Following incubation overnight at 30°C, colonies sensitive to hygromycin were re-streaked onto EMM+L plate and replica plated onto EMM plates to confirm loss of the plasmid. The leucine auxotrophic colonies were used for subsequent experiments.

### SSA assay growth conditions and genetic colony assay

Logarithmically growing cells grown at 30°C in EMM+L were pelleted and re-suspended in pre-warmed EMM+LH (P*_urg1lox_* OFF) or EMM+LH supplemented with uracil at 0.25 mg/ml (P*_urg1lox_ ON*) and incubation continued at 30°C. At the indicated time points 500 cells were plated on EMM+LH agar and grown at 30°C until colonies appeared. The resulting colonies were replica plated onto EMM+L agar, grown at 30°C and the percentage of histidine auxotrophic colonies calculated.

### Preparation of total cell extract and Western blot analysis

Preparation of cell extracts for SDS-PAGE and Western blotting was performed as previously described [7]. Mouse monoclonal anti-GFP antibody (Roche) was diluted 1∶5,000, rabbit anti-PCNA antibody (Gift: A. Lehmann) was diluted 1∶2,000 and mouse monoclonal anti-HA (Santa Cruz Biotechnology) diluted 1∶2,500. As a loading control, mouse monoclonal anti-tubulin antibody (diluted 1∶10,000; Sigma) was used.

### 2D gel electrophoresis

Cells were grown in EMM media supplemented with adenine and leucine (EMM+AL) at 30°C to a density of approximately 1×10^7^ cells/ml and 1.25×10^9^ cells harvested by centrifugation. P*_urg1lox_-rtf1* expression was induced by the addition of uracil at 0.25 mg/ml (P*_urg1lox_ ON*). Chromosomal DNA was extracted using standard procedures, embedded in agarose plugs and digested using 30 units of A*se*I. Digested chromosomal DNA was analysed by 2D gels [27], using 0.35% and 0.9% agarose for the first and second dimensions, respectively. Replication intermediates were visualized using pCen (centromere proximal to the *ura4* gene) as a probe. Probe pCen template DNA was amplified from total genomic *S. pombe* DNA using primers P31 and P32. Autoradiography was performed using a storage phosphor screen/Storm PhosphorImager system.
